# Performance evaluation of Truenat MTB and Truenat MTB-RIF DX assays in comparison to gene XPERT MTB/RIF ultra for the diagnosis of pulmonary tuberculosis in Uganda

**DOI:** 10.1186/s12879-024-09063-z

**Published:** 2024-02-13

**Authors:** Willy Ssengooba, Achilles Katamba, James Sserubiri, Derrick Semugenze, Abdunoor Nyombi, Raymond Byaruhanga, Stavia Turyahabwe, Moses L. Joloba

**Affiliations:** 1 Department of Medical Microbiology, Makerere University, Kampala, Uganda; 2 Makerere University Lung Institute, Kampala, Uganda; 3 Biomedical Research Center, Makerere University, Kampala, Uganda; 4 Department of Medicine, School of Medicine, Clinical Epidemiology and Biostatistics Unit, Makerere University, Kampala, Uganda; 5 Uganda TB Implementation Research Consortium, Kampala, Uganda; 6 Ministry of Health, National Tuberculosis, and Leprosy Programme, Kampala, Uganda

## Abstract

**Background:**

The World Health Organization endorsed Truenat MTB rapid molecular assay in 2020 and recommended additional in-country evaluation studies before uptake. We evaluated the accuracy and operational feasibility of Truenat MTB assay (Truenat) in comparison with GeneXpert Ultra and culture.

**Methods:**

In a cross-sectional study of 250 presumptive TB patients, participants were requested to provide a sputum sample on the day of their visit to the clinic. The sputum sample was homogenized and a portion was tested using GeneXpert Ultra as per the routine standard procedure and the other portion was tested using Truenat assay at the clinic laboratory. The second sample portion was processed for Concentrated Fluorescent smear Microscopy (CFM), LJ, and MGIT cultures. Truenat sensitivity and specificity were compared to GeneXpert Ultra and culture. Test performance characteristics and operational feasibility assessment data through interview of the study laboratory staff were also collected and summarized as proportions and percentages.

**Results:**

Of the 250 participants recruited in the study, the sensitivity and specificity of Truenat was n/N (%, 95%CI); 66/82 (80.5, 70.2–88.4) and 156/159 (98.1, 94.5–99.6) when compared with Ultra, 50/64 (89.3, 66.0-87.4) and 166/180 (92.2, 87.2–95.6) when compared with LJ, 58/71 (81.7,70.7–89.8) and 131/138 (94.9, 89.8–97.9) when compared to MGIT culture and 59/73 (80.8, 69.9–89.1) and 159/169 (94.1,89.3–97.1) when compared to LJ and/or MGIT culture. The sensitivity of Truenat was lower, 14/23 (60.9, 40.6–82.8) among smear-negative compared to 45/50 (90.0, 78.1–96.6) among smear-positive participants but not different by HIV status. There were no special training needs especially among laboratory personnel with previous GeneXpert /molecular test experience, 19/242 (7.8%) error/invalid, and 12 (17,4%) uninterpretable/indeterminate results mainly for rifampicin resistance determination. However, there were 3 (3.5%) of GeneXpert Ultra indeterminate results.

**Conclusion:**

Among presumptive TB patients in Uganda, the Truenat assay has high sensitivity and specificity. The Truenat assay has acceptable operational feasibility attributes when compared with the GeneXpert Assay.

**Supplementary Information:**

The online version contains supplementary material available at 10.1186/s12879-024-09063-z.

## Background

Tuberculosis (TB) remains the number one cause of death globally attributable to a curable infectious agent [1]. Over 95% of new TB cases and deaths occur in developing countries. In 2021, Uganda with an estimated population of 46 million people, had a TB incidence of 199(119–298)/100,000 population and an MDR/RR-TB incidence of 63 (38–98)/100,000 population. Only 69% of the TB cases were tested with a rapid diagnostic test and 75% were tested for rifampicin resistance at the time of diagnosis [1]. Culture, while reliable, takes weeks to obtain a result, smear microscopy is still the most common immediate diagnostic in most countries, but only detects 45% of TB infections [2]. Novel molecular rapid tests provide an avenue for immediate and accurate TB diagnosis [3].

The GeneXpert® MTB/RIF Assay and currently the 3rd generation GeneXpert Ultra (Cepheid, USA) is a rapid, automated molecular test that can detect both TB and rifampicin resistance within about two hours with minimal hands-on time.

The WHO endorsement of the GeneXpert assay in 2010 and in 2017 for a more sensitive GeneXpert Ultra (Ultra) has revolutionized TB diagnosis [4]. As yet, GeneXpert is the only Nucleic Acid Amplification Test (NAAT) with unconditional endorsement from WHO. By increasing access to more innovative NAATs, end users would benefit from options for diagnostic tests [5]. Since 2020, there is an array of NAAT tests with conditional endorsement from the WHO, which require additional evaluation data in order to receive the full endorsement. The Truenat™ MTB plus and Truenat™ MTB tests (Truenat: Molbio Diagnostics, Bangalore, India) are the first to mature in this pipeline, potentially providing the opportunity to address the much-needed demand of rapid diagnostic tests to detect TB. However, there are still little implementation studies with sufficient clinical data to support country uptake and rollout. The Truenat assays® chip-based assay are an alternative to the GeneXpert assay that have been developed for use in primary health care facilities. There are two cartridge-based assays: one for TB detection (Truenat™ MTB or Truenat™ MTB plus) and a second (reflex: Truenat™ MTB-RIF Dx) assays to test any positive samples for rifampicin resistance, collectively referred to as Truenat in this study [6]. Uganda planned to rollout Truenat in areas with GeneXpert assy implementation challenges and as complementary backup in case of supplies stock out among other challenges. In this evaluation study, we aassessed the sensitivity and specificity of Truenat assays in raw sputum compared to the WHO-endorsed GeneXpert® MTB/RIF Ultra using culture as the reference standard. We also assessed the operational feasibility of Truenat assays compared GeneXpert Ultra to inform rollout of the test.

## Materials and methods

This was a cross-sectional study to determine the accuracy and operational feasibility of the Truenat assays among individuals with symptoms of pulmonary TB (PTB) in comparison to Gene Xpert® MTB/RIF Ultra assay and in comparison with a rigorous culture-based gold standard. Gene Xpert® MTB/RIF Ultra assay results were used by the clinicians for the care of patients, however; the results of the investigational Truenat assays were not used for clinical care and were not provided to clinicians or participants. Enrolment took place at the Out Patients Departments (OPDs) of Kampala Capital City Authority (KCCA) Health facilities including; Kisenyi Health Center IV, Kawaala Health Center IV, Kitebi Health Center III, and Kiswa Health Center III, and at the Namungoona Orthodox Hospital.

The study’s target enrolment was 250 analysis-eligible participants. Eligible individuals were male and female, aged 18 years and above who had symptoms consistent with pulmonary TB (Fever, Cough for > 2 weeks, unexplained weight loss, night sweats and chest pain) presenting to participating health centers. Eligible participants were recruited at the TB laboratory on delivery of sputum samples. The study procedures were explained to presumptive TB patients and those who provided a written informed consent to participate in the study were enrolled. A study questionnaire was administered to collect patient demographic and clinical information. HIV-positive individuals and HIV-negative individuals were included in this study.

### Laboratory procedures

**Fig. 1:**
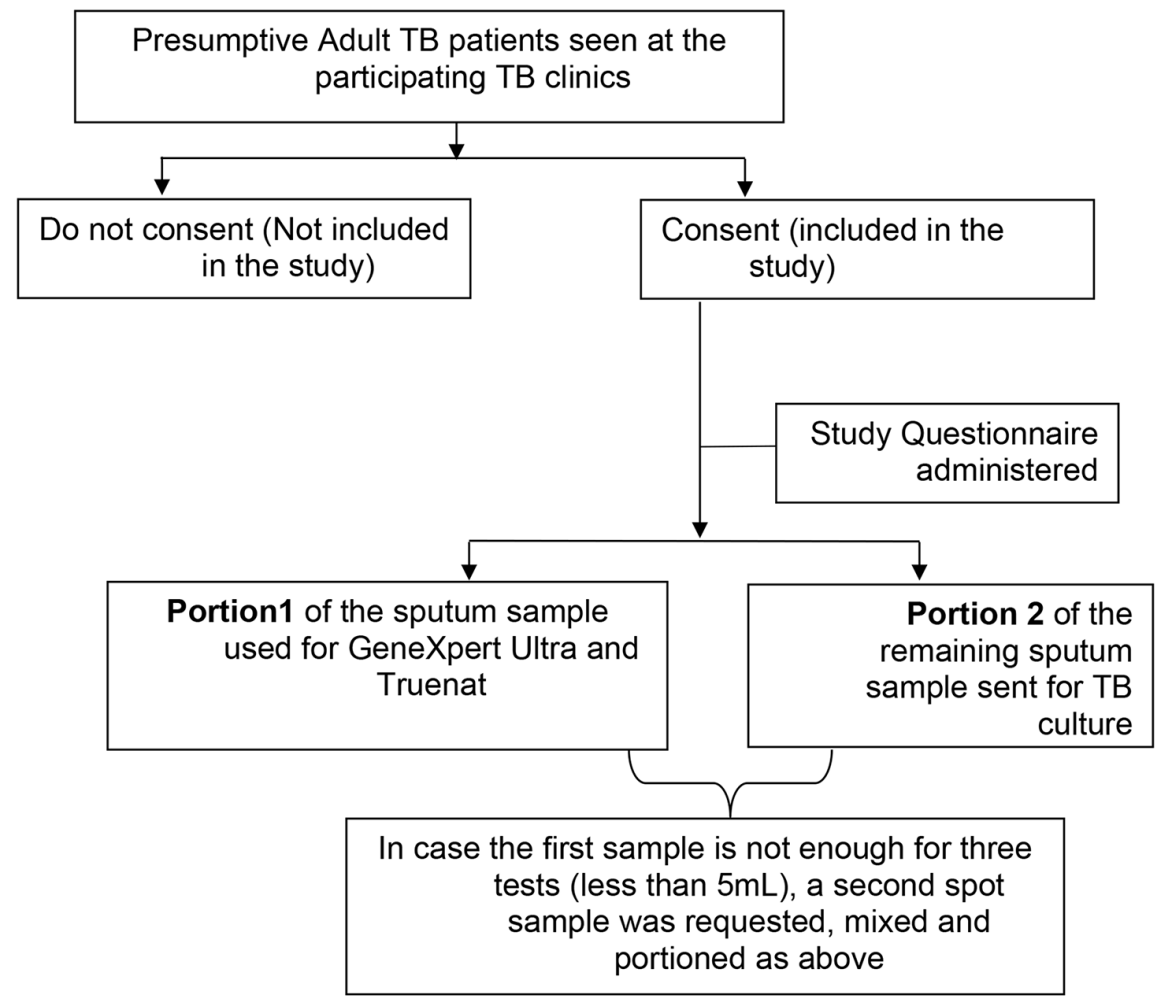
Schematic of study design and recruitment plan

Sputum samples were mixed and divided into two portions. Following no sampling order and to avoid sampling bias, portion 1 was tested using GeneXpert Ultra as per the routine standard procedure. This was also tested using Truenat MTB assay and if positive reflexed to Truenat™ MTB-RIF Dx for rifampicin resistance testing following manufacturer’s instructions. Portion 2 of the sample was sent to the College of American Pathologist (CAP) ISO15189 accredited Mycobacteriology (BSL-3) laboratory at School of Biomedical Sciences, Makerere University for TB culture (Fig. 1).

For TB culture, samples were decontaminated with *N*-Acetyl-L-Cysteine–Sodium Hydroxide (NALC-NaOH: final concentration 1.5%) for 15 min and diluted with phosphate-buffered saline (PBS, pH 6.8). The mixture were centrifuged at 3000 g for 15 min and the pellet was resuspended in 1.5 ml of PBS. A concentrated smear and Löwenstein–Jensen (LJ) and Mycobacterium Growth Indicator Tube (MGIT) culture were performed according to standard operating procedures (SOPs) of the Mycobacteriology (BSL-3) Laboratory. Sample with culture growth having Ziehl-Neelsen (ZN) positive smear and MPT64 antigen positive were considered to have Mycobacterium Tuberculosis Complex (MTBC) otherwise samples with ZN positive but MPT64 antigen negative were considered to have growth due to Non-Tuberculous Mycobacteria (NTM). Cultures that were ZN smear-negative but with growth on blood agar were considered contaminated.

### Operational feasibility assessment

After obtaining, a verbal consent data were collected through direct observation of study laboratory staff as they perform study laboratory procedures for Truenat. We also conducted interviews with study laboratory staff using a semi-structured questionnaire. Interviews of the laboratory staff was done for two personnel with previous GeneXpert experience and two personnel without. Those without prior GeneXpert experience, were trained in both GeneXpert and Truenat tests and those with, only trained for Truenat, all for one day. Demonstration samples were used in both training and independent testing using the study Standard Operating Procedures (SOP) before Operational feasibility included; robustness of reagents and equipment in relation to temperature, dust and power irregularities, ease of use, contamination events and number of invalid results. For this evaluation study, the maximum acceptable indeterminate rate was set at 10%.

### Statistical analysis

Descriptive data were analyzed as proportions and percentages. For diagnostic accuracy, the samples, which were invalid or had an error on Truenat after two repeats were excluded from the analysis. For comparison of Truenat and GeneXpert ultra samples, which had invalid, and error results were excluded. Furthermore, for samples which had contamination and/or NTM on culture were excluded from culture comparison (Supplement 1). The sensitivity and specificity were calculated in reference to GeneXpert Ultra, LJ culture, MGIT culture, and any culture. Additional analyses were stratified by smear microscopy, HIV status, history of TB, and history of smoking. Analysis for specificity included all Non-TB participants as per GeneXpert ultra or culture. The sensitivity of a test was defined as the number of index test positives divided by the number of comparator test positive and the specificity as the number of test negatives divided by all Non-TB). Confidence intervals for all sensitivity and specificity estimates for the molecular tests and the reference standard were calculated using binomial proportion confidence intervals. Results of operational feasibility were presented as frequencies of occurrences or as reported by the study laboratory staff interviewed.

## Results

Of the 250 participants recruited in the study, the majority were male 141 (56.4%), 168 (67.2%) were aged 24–44 years, and 248 (99.2) reported cough. A total of 109 (43.6%) were HIV-positive and 50 (20.0%) were previously treated for TB, Table 1.

**Table 1:** Clinical and demographic characteristics of the study participants

Parameter (*N* = 250)	n (%)
**Gender**	
Male	141(56.4)
Female	108 (43.2)
Missing	1(0.4)
Age Category (years)	
Median age (IQR)	32.5(30–35)
18–24	43 (17.2)
25–44	168 (67.2)
45–69	34 (13.6)
> 60	5 (2.0)
**TB Symptoms**	
Cough	248 (99.2)
Fever	137 (54.8)
Weight loss	143 (57.2)
Night sweats	128(51.2)
Chest pain	126 (50.4)
Previously diagnosed	50 (20.0)
**HIV status (246)**	
Positive	109 (43.6)
On ART	79 ( 31.6)
**History of smoking (249)**	
Never smoked	177 (71.1)
Ever smoked	72 (28.9)
Current smoker	31 (43.1)
Stopped smoking	41 (56.9)

^ART= Antiretroviral Therapy, TB= Tuberculosis, IQR− Interquartile Range, %= percentage^

### Positivity rates for Mycobacterium tuberculosis by test method

Of the 250 participants, smear microscopy was positive among 57 (22.8%) participants. The smear grading was, scanty 9 (15.8%), 1 + 15 (26.3%), 2 + 18 (31.6%), and 3 + was 15 (26.3%). Only 249 participants were tested using Ultra, for whom, 84 (33.7%) had MTB detected, all with no rifampicin-resistance (RR) detected, and 3 (3.5%) had indeterminate results. The semi-quantitative grading for MTB detected is summarized in Table 2. Truenat had test results for only 242 participants for whom 69 (28.5%) had MTB detected, 1 (1.5%) rifampicin-resistance detected, and 12 (17.4%) had indeterminate results. Only 245 participants had LJ culture results, of whom 57 (23.3%) were positive for MTB and 1 (0.4%) was contaminated. All participants had MGIT culture results, of whom, 72 (28.8%) were positive for MTB, 21 (8.4%) were contaminated and 14 (5.6%) grew NTM, Table 2.

**Table 2:** Positivity rates for Mycobacterium tuberculosis by test method

Test/Results	n (%)
**TrueNat (*N*** = 242)	
MTB detected	**69 (28.5)**
Rifampicin Resistance Not Detected	56 (81.2)
Rifampicin Resistance detected	1 (1.5)
Indeterminate	12 (17.4)
**GeneXpert Ultra (** ***N*** ** = 249)**	
MTB detected	**84 (33.7)**
Trace	2 (2.4)
Very low	10 (11.7)
Low	25 (29.4)
Medium	20 (23.5)
High	28 (32.9)
Rifampicin Resistance Not Detected	81(96.4)
Rifampicin Resistance detected	0
Indeterminate	3 (3.5)
**Concentrated smear microscopy (** ***N*** ** = 250)**	
Positive	57 (22.8)
Scanty	9 (15.8)
1+	15 (26.3)
2+	18 (31.6)
3+	15 (26.3)
**LJ Culture (** ***N*** ** = 245)**	
MTBC positive	**57 (23.3)**
Contaminated	1 (0.4)
**MGIT Culture (** ***N*** ** = 250)**	
MTBC positive	**72 (28.8)**
Contaminated	21 (8.4)
NTM	14 (5.6)

^LJ= Lowenstein Jensen, MGIT= Mycobacterial Growth Indicator Tube, % percentage, NTM= Non-Tuberculous Mycobacteria, MTBC= Mycobacterium Tuberculosis Complex^

### Diagnostic accuracy of Truenat assay compared to different bacteriological standards

The sensitivity and specificity n/N (%, 95%CI), of Truenat were 66/82 (80.5, 70.2–88.4) and 156/159 (98.1, 94.5–99.6) when compared to GeneXpert Ultra. However, when compared to LJ culture the sensitivity and specificity of Truenat were 50/64 (89.3, 66.0-87.4) and 166/180 (92.2, 87.2–95.6) while when compared to MGIT culture it was 58/71 (81.7,70.7–89.8) and 131/138 (94.9, 89.8–97.9), respectively. If any culture was used, the sensitivity and specificity of Truenat assay were 59/73 (80.8, 69.9–89.1) and 159/169 (94.1, 89.3–97.1) respectively, Table 3.

**Table 3:** Diagnostic accuracy of Truenat assay compared different bacteriological tests as reference comparator

Reference comparator (N)	Sensitivity n/N (%, 95%CI)	Specificity n/N (%, 95%CI)	PPV n/N (%, 95%CI)	NPV n/N (%, 95%CI)
**GeneXpert Ultra**	66/82 (**80.5, 70.2–88.4)**	156/159 (**98.1, 94.5–99.6**)	66/69 (95.6,87.8–99.1)	156/172 (90.7, 85.3–94.6)
**LJ Culture**	50/64 (**89.3, 66.0-87.4**)	166/180 (**92.2, 87.2–95.6**)	50/64(78.1, 66.0-87.4)	166/172 (96.5, 93.5–98.7)
**MGIT culture**	58/71 (**81.7, 70.7–89.8**)	131/138 (**94.9, 89.8–97.9**)	58/65 (89.2,79.0-95.5)	131/144 (91.0, 85.0-95.1)
**LJ and/or MGIT**	59/73 (**80.8, 69.9–89.1)**	159/169 (**94.1, 89.3–97.1**)	59/69 (85.5, 74.9–92.8)	159/173 (91.9, 86.7–95.5)

^LJ= Lowenstein Jensen, MGIT= Mycobacterial Growth Indicator Tube, CI = Confidence Interval, % percentage, PPV=Positive Predictive Value, NP = Negative Predictive Value^

Among participants with any of culture results, the sensitivity of the Truenat assay was lower, 14/23 (60.9, 40.6–82.8) among smear-negative compared to 45/50 (90.0, 78.1–96.6) among smear-positive participants. The sensitivity was not different by HIV-status, Table 4. The factors associated with Truenat assay positivity (AdjOR (p-Value, 95%CI) were; History of smoking 2.21 (0.01, 1.21–4.05) and being HIV-positive 0.52 (0.03, 0.28–0.95).

**Table 4:** Sensitivity and specificity of Truenat assay by smear and HIV status by any culture as a reference standard

Patient category	Truenat Sensitivity n/N (%, 95%CI)	Truenat Specificity n/N (%, 95%CI)
**Smear status**		
Smear negative	14/23 (60.9, 38.5–80.2)	155/164 (95.1, 90.5–97.8)
Smear positive	45/50 (90.0, 78.1–96.6)	4/6(66.7, 22.2–95.6)
**HIV status**		
HIV-positive	16/20 (80.0, 56.3–94.2)	81/85 (95.3, 88.3–98.7)
HIV- negative	42/51 (82.3, 69.1–91.6)	77/83 (93.0, 84.9–97.3)
**Previous history of TB**		
History of TB	9/12 (75.0, 42.8–94.5)	31/35 (88.6, 73.2–96.7)
No history of TB	50/61(82.0, 70.0–90.6)	128/134 (95.5, 90.5–98.3)
**History of smoking**		
Never smoked	35/45 (77.8, 62.9–88.7)	121/127 (95.3, 90.0–98.2)
Ever smoked	24/28 (85.7, 67.3–95.9)	37/41 (90.2, 76.8–97.2)
Stopped smoking	14/16 (87.5, 61.6–98.4)	19/22 (86.4, 65.0–97.0)
Current smoker	11/13 (84.6, 54.5–98.0)	17/18 (94.4, 72.7–99.8)

### Operational feasibility of Truenat MTB assays

The training needs for Truenat assay were considered less for laboratory personnel with previous GeneXpert /molecular test experience, and was considered to be learned within two 2-days for those without previous GeneXpert /molecular test experience. This is similar to what is required for GeneXpert test training. The sample processing steps were considered easy since Truenat only has two steps i.e. extraction and amplification, which are also automated. The recording and reporting needs were also considered less skills demanding and similar to those required for GeneXpert Ultra test. This was because both test methods give print-out of interpreted results automatically. The proportion of error/invalid was 19/250 (7.6%) whereas for uninterpretable/indeterminate results was found to be 12 (17.4%), mainly for determination of rifampicin resistance. However, there were 3 ( 3.5%) GeneXpert Ultra indeterminate results. The average Truenat results Turn Around Time (TAT) was 2:30 h for negative and 1:30 h for positive results. The TAT did not include time for reflex testing for rifampicin resistance since this only applies for cases when *M. tuberculosis* was detected. The workflow for Truenat was reported to be having few additional steps compared to those while using GeneXpert Ultra, mainly related to the extraction step.

## Discussion

Our study findings among presumptive TB patients in Uganda show that the Truenat assay had a sensitivity of 81% (59/73) and specificity of 94% 159/169. This was lower than the sensitivity of 93.1% (68/73) for GeneXpert Ultra in this study population. However, the specificity of Truenat among culture negative patients was slightly lower, 90.9% (10/176) for GeneXpert ultra compared to Truenat. Molecular tests tend to agree more with molecular reference standards and there was no difference in sensitivity of Truenat assay, 80.5% (66/82) when GeneXpert was used as a reference standard compared to 80.8% (59/73) when LJ and or MGIT was used as a reference standard. The majority of Truenat negative but GeneXpert ultra positive (16/83) were 2 (100%) trace, and 6/10 (60%) very low GeneXpert ultra semi-quantitative grades. Truenat assay attained similar sensitivity 80%, among culture-positive people living with HIV. However, Truenat assay sensitivity was 61% (14/23) and 75% (9/12) among previously treated culture-positive participants respectively.

Our study documented comparable Truenat sensitivity done on the same sample under primary health care to that documented in a multicenter diagnostic accuracy study [7]. However, slightly lower sensitivity of Truenat MTB Plus has been documented compared to other previous studies [8, 9] possibly and partly attributed to the fact that these studies analyzed two sputum samples rather than one spot sample in our study, which is routinely done in Uganda, and were largely not done in primary health care centers. In addition, these previous studies had a different composition of smear negative but culture positive individuals than in our study. The WHO guidelines recommending the use of Truenat in adults with signs and symptoms of TB makes all Truenat positive patients eligible to start TB treatment [4]. However, these WHO guidelines makes a conditional recommendation with uncertainty of Truenat performance among PLHIV. Notably for our study, 40% (4/10) Truenat negative but culture positive were HIV-positive. Of this 2/4 were detected by LJ culture whereas all four were positive on MGIT culture.

The overall specificity of the Truenat assay was high in all aspects of comparisons. The specificity of Truenat was 94% (159/169) among culture-negative patients. This was comparable to the specificity documented in the previous studies [7–9]. Culture is considered the gold standard for TB diagnosis; however, there remain unresolved discordances when compared with molecular diagnostics. For example, the specificity for Truenat was higher, 98% compared to 94% for Genexpert when LJ and/or MGIT were used as reference comparators respectively. Some of the reasons could be that molecular tests are detecting DNA from previous episodes of tuberculosis disease. In our study, we found the specificity of Truenat among previously treated TB patients to be 88% compared to 96% among those with no history of TB treatment. This may suggest that the 10 participants who were Truenat positive but culture negative either had TB DNA from none viable MTB or had very low bacterial load which may have been reduced further by the processing for the culture to be positive. Nine of the ten Truenat-positive but culture-negative participants were Genexpert ultra positive with grades; very load (*n* = 2), low (*n* = 6), and high (*n* = 1). Based on this evaluation for specificity, these were considered Truenat false positives compared to a culture-based reference standard. This was not different from what was observed when GeneXpert ultra was compared to a culture-based reference standard. Majority of GeneXpert Ultra false postives 14/16 (87.5%) were of grades between low and trace of whom 50% were previously treated for TB. Previous studies have considered participants who are positive on a molecular test but culture-negative to possibly be having TBDNA resulting from the previous disease episode [10]. In our study only 4/10 Truenat positive but culture negative participants had history of previous TB. This observation is also seen in the previous studies comparing culture and molecular tests, which is attributed to a false positive test due to DNA resulting from dead bacilli [9, 11–13]. Management of such patients is dependent on the clinician’s decision combined with other clinical evaluation in line with TB disease. However, following the recommendation of the WHO for GeneXpert ultra, Truenat, or culture as standard tests for TB diagnosis, these should be started on TB treatment [4]. Indeed, all GeneXpert ultra and/or culture-positive TB patients in our study were started on TB treatment.

On the evaluation of Truenat in line with operational feasibility, we found the test to be a good point-of-care test and feasible for roll-out in health care centers. Technically the test is less demanding in terms of skills needed from the laboratory technician and required at most 2-days of training. Truenat had a high proportion of error/invalid 19/250 (7.6%) which gave valid results on repeat. Also, there was a high rate of uninterpretable/indeterminate results 17.4%, mainly for rifampicin resistance determination. These have also been documented in previous studies and require consideration from the manufacture to be reduced to a tleast below 5% [7, 13]. Truenat has been validated on multiple pathogen testing, although more pathogens and disease conditions are validated for GeneXpert. Implementation of Truenat could be done in a complementary way, in that, sites with Truenat could have access to GeneXpert site for possible referral testing in case Truenat is down or in cases of supplies tsock-out and vice versa.

Our study had several strengths. The index test and its comparator (GeneXpert ultra) were performed in primary health care facility laboratories, where the Truenat test is intended to be used, hence depicting the real-world results. The reference comparator, sputum culture, were performed in the College of American Pathologist (CAP:ISO15189) accredited Mycobacteriology (BSL-3) laboratory. Performing the Truenat test, GeneXpert ultra and culture on the same sample, eliminated potential sample handling bias that makes our findings stronger for test performance comparison. We compared the performance of Truenat with GeneXpert ultra ( a more advanced version of GeneXpert test), which is not the case with most of the previous Truenat evaluation studies [7–9, 14]. Our study had a bigger sample size of PLHIV (44%) which makes the results more generalizable to other high HIV/TB burden settings. We had a robust reference standard of solid and liquid culture. However, our study also had some limitations. The testing was based on one sputum sample which may have reduced the ability to conclusively identify a TB case, however, this mainly reflected what happens in routine where presumptive TB patients are tested on GeneXpert ultra using one sputum sample. Not all participants had Truenat test performed (242/250) however, these were few participants to affect our study findings.

## Conclusions

The findings of our study show that Truenat test has acceptable sensitivity and specificity compared to GeneXpert Ultra and culture. These remain acceptable even among people living with HIV as per WHO recommendation. Additional evaluation in the implementation mode to assess the performance among smokers as well as those who are Truenat positive but culture negative as well as the high indeterminate results for rifampicin resistance determination are needed.

### Electronic supplementary material

Below is the link to the electronic supplementary material.

Supplementary Material 1

## Data Availability

All data generated or analysed during this study are included in this published article and its supplementary information files (Supplement 1).
